# Agricultural and Ecological Resources Safeguarded by the Prevention of Wild Pig Population Expansion

**DOI:** 10.3390/biology13090670

**Published:** 2024-08-29

**Authors:** Colin Jareb, Kim M. Pepin, Ryan S. Miller, Sarah Sykora, Stephanie A. Shwiff, Sophie C. McKee

**Affiliations:** 1 National Wildlife Research Center, United States Department of Agriculture, Animal and Plant Health Inspection Service, 4101 Laporte Avenue, Fort Collins, CO 80521, USAsarah.sykora@usda.gov (S.S.); sophie.mckee@colostate.edu (S.C.M.); 2 Center for Epidemiology and Animal Health, United States Department of Agriculture, Veterinary Services, Fort Collins, CO 80526, USA; ryan.s.miller@usda.gov; 3 Department of Economics, Colorado State University, Fort Collins, CO 80523, USA

**Keywords:** agricultural resources, economic impact, feral swine, invasive species, population dynamics, *Sus scrofa*, United States, wild pigs

## Abstract

**Simple Summary:**

The spread of invasive wild pigs across the United States has been successfully slowed due to the establishment of a national management program. In this paper, we study the effectiveness of the national program by modeling the spread of wild pigs in the absence of intervention. Further, we use the models to assess the value of resources safeguarded from the threat of wild pigs. Our findings indicate that, on average, invasive wild pigs were prevented from spreading to 724 counties, and USD 40.2 billion in resource value was safeguarded over the first eight years of the program. Continuation of the program will deliver additional benefits and further research is critical to understand its comprehensive economic effects.

**Abstract:**

Wild pigs (*Sus scrofa*) are one of the most destructive invasive species in the US, known for causing extensive damage to agricultural commodities, natural resources, and property, and for transmitting diseases to livestock. Following the establishment of the National Feral Swine Damage Management Program (NFSDMP) in 2014, the expansion of wild pig populations has been successfully slowed. This paper combines two modeling approaches across eight separate models to characterize the expansion of wild pig populations in the absence of intervention by the NFSDMP and forecasts the value of a subset of resources safeguarded from the threat of wild pigs. The results indicate that if wild pigs had continued spreading at pre-program levels, they would have spread extensively across the US, with significant geographic variation across modeling scenarios. Further, by averting the threat of wild pigs, a substantial amount of crops, land, property, and livestock was safeguarded by the NFSDMP. Cumulatively, between 2014 and 2021, wild pig populations were prevented from spreading to an average of 724 counties and an average of USD 40.2 billion in field crops, pasture, grasses, and hay was safeguarded. The results demonstrate that intervention by the NFSDMP has delivered significant ecological and economic benefits that were not previously known.

## 1. Introduction

Initially introduced to the United States in the 1500s, wild pigs (*Sus scrofa*), also known as feral swine, wild swine, wild boar, or feral hogs [1], are a successful invasive species [2]. For this study, the collective term wild pig will be used [1] unless it is appropriate to use one of the common names discussed above. Native to Europe, Asia, and Northern Africa [3], wild pigs have been repeatedly introduced into the United States, with populations observed in at least 38 states [4]. Further, high reproductive rates and generalist habitat use have led to transient population dynamics [5] and highly variable geographic rates of population spread [6,7].

The literature on the economic damages caused by the spread of wild pigs is growing. In a survey of producers of six crops in 11 states, McKee et al. [8] estimate that USD 203 million of crop production was lost in 2022. Using a survey of producers of additional crops across 12 states in 2019, McKee et al. [9] estimate crop losses of USD 272 million. Boughton et al. [10] studied the effects of wild pig rooting and concluded that damage to pastureland and loss of forage generated regional losses greater than USD 2 million annually. Further, Didero et al. [11] provide examples of a wide range of methodologies used to produce damage estimates. Lastly, Vercauteren et al. [12] contribute comprehensive treatment on a variety of issues regarding the extent of knowledge about wild pig control and damage.

As a result of the outlined population dynamics and increasing damage, US Congress appropriated funds in 2014 to establish the National Feral Swine Damage Management Program (NFSDMP). The objectives of the program include operational population reduction, such as removal and eradication, cooperative research, and outreach and communication campaigns. The impacts from wild pig damage have primarily been felt in areas with large wild pig populations, such as the Southeastern United States and California. As a result of this geographic concentration, the NFSDMP’s resources have been focused on mitigating damage in a few states and eliminating wild pigs in other states. In states with significant invasive wild pig populations, surveys have been conducted to estimate the extent of damage to corn, wheat, peanuts, rice, sorghum, soybeans, cotton, hay, almonds, pecans, melons, sugarcane, sweet potatoes, and berries in these areas [8,9].

Focusing on invasive wild pig population control, the NFSDMP has eliminated feral swine from 11 states [13], reduced spread rates [5], and prevented the spread of wild pigs to regions with no contemporary or previous wild pig populations [7]. By reducing the spread of wild pigs, the introduction of the NFSDMP has alleviated the threat of damage to resources including crops, land, property, and livestock.

However, little is known about the damage prevented by management in areas where pig populations would have been established and grown in the absence of intervention [7]. Estimating the extent of wild pig geographic spread prevented by NFSDMP activities allows for the evaluation of resources safeguarded by management. In this paper, we use two ecological modeling frameworks, a dynamic occupancy approach [14,15] and boosted regression trees [16] to forecast the spread of wild pigs between 2014 and 2021 in the absence of any intervention by the NFSDMP. Further, we utilize the spread forecasts to quantify the primary resources safeguarded from the threat of wild pigs during this period.

Analyzing the full extent of damages caused by the spread of wild pigs requires information on population densities and the degree to which wild pigs damage resources. However, the models forecasting the spread of wild pigs focus on predicting the share of counties occupied by wild pigs without providing predictions on density. Little is known about the relationship between the presence and density of wild pigs and the damage they cause [12], nor of the extent of damage caused by wild pigs to a variety of other crop resources and to land resources, such as wetlands [11]. For these reasons, it has been intractable to ascertain the full economic impact of wild pig damage avoided by introducing the NFSDMP. Therefore, our main objective was to quantify the economic value of resources safeguarded by a national-scale wild pig damage management program. We estimate the total resource acreage (including agricultural crops, pasture, and wetlands) and livestock inventories safeguarded from the presence of wild pigs by linking forecasted geographic spread of wild pigs in the absence of NFSDMP to the value of selected commodities in those areas where spread was prevented. We then estimate a dollar value for a subset of the resources for which annual revenue per acre is available.

Our results find that from 2014 to 2021, the NFSDMP has successfully prevented the spread of wild pigs to additional counties. Further, our analysis demonstrates that significant acreage and value in field crops, pasture, and grasses has been successfully safeguarded by the NFSDMP. These results indicate that the additional benefits generated by implementing the NFSDMP in areas where wild pigs have not been observed are significant and should be accounted for when evaluating the benefits of the program. Our framework can be used to evaluate the value of resources safeguarded by a national damage management program.

## 2. Materials and Methods

We modeled the economic consequences of preventing the spatial spread of wild pigs using an ensemble approach [17]. We forecast the spread of wild pigs using two approaches; boosted regression tree modeling with step-ahead forecasting from these models and a dynamic occupancy approach. For each approach, we further include a variety of covariates, simulating each model using four distinct parameterizations. This yields a total of eight forecasts that predict the spread of wild pigs in the absence of NFSDMP intervention. We quantify the resources safeguarded by containing the spread of wild pigs by using the total acres or head of a resource and the proportion of the county forecasted as occupied by wild pigs. Further, we combine this amount with the economic value of the respective resource to quantify the value of resources safeguarded. The two modeling frameworks were implemented at the county and watershed scales. All final model predictions are provided at the county scale annually and provide both the presence/absence and proportion of the county occupied by wild pigs. We follow previous work [7,18] by including a variety of covariates to predict the rate of spatial spread.

### 2.1. Data and Processing

We modeled the geographic expansion of wild pigs using annual county-level and watershed-level mapping of wild pig presence/absence [6,7,18]. These data were expressed as a county area occupied by wild pigs in km^2^ and are a proportion of the county occupied by wild pigs. We subdivided the data into counties where there have been wild pigs at least once during 2009–2013 (‘transient counties’, *N* = 926) and those where there have always been wild pigs during 2009–2013 (‘persistent counties’, *N* = 376) because we expected spread rates and covariate effects to differ in these two situations. For estimating the pre-NFSDMP spread rates, first, we estimated annual spread rates for the 5 years immediately prior to the start of NFSDMP (2009–2013) by subtracting the km^2^ area or proportion occupied in the current year from that in the previous year. Then, we took the mean of these annual spread rates per county to generate the average pre-program spread rates to estimate covariate effects.

Watershed level response data were obtained using the following procedure. Polygons representing the observed distribution of wild pigs were aggregated to 12-digit watershed boundary data using methods described in Miller et al. [5] resulting in the presence/absence of wild pigs by 12-digit watersheds. These 12-digit watersheds were assumed to represent discrete populations. The number of the invaded and total number of 12-digit watersheds within each coarse scale 8-digit watershed was generated and used as a response variable.

We use data from the National Feral Swine Mapping System to estimate the rate of spread of wild pigs in the contiguous United States. These data have been compiled annually since 2008. Each year, wildlife professionals in state wildlife resource agencies and the United States Department of Agriculture report polygons (polygons are drawn by technicians and vary in size based on extent of observed wild pigs) representing their opinion of the geographic extent of established wild pig populations to the National Feral Swine Mapping System. The definition of pig presence in this methodology includes populations present for two or more years with evidence of reproduction. The polygons are then processed to determine the proportion of each county that has wild pigs.

There are two major sources of error in the data that are difficult to account for because we did not have the requisite data: (1) error between the perception of where wild pigs are when experts draw the polygons and the true distribution of wild pigs; (2) differences in the reporting methodology over time and across states in terms of how detailed the polygons were and how much observational effort was allotted to their reporting. Because of these data limitations with interannual variation, we estimate the pre-program spread rate from 2009 to 2013 as an average rate across those 5 years. This was performed by first calculating the county-level annual rate for each year (2009–2013) as the difference in proportion occupied for each county between year_t_ − year_t−1_, which ranged from −1 to 1. We also investigated models of the spread rates per km^2^ because counties varied widely in their size. To estimate this second response variable, we multiplied the proportion differences by the county area to model the rate spread in km^2^ and account for differences in county size. This response distribution ranged [−542.4, 3165.8], and as with the difference in proportion distribution, the change in km^2^ response probability distribution was characterized by a high density near 0, a short tail on the negative-value side, and a long tail on the positive-value side. We investigate models of the change in proportions as well as models of the change in km^2^ occupied.

We obtain crop, fruit, vegetable, grass, and pasture resource data at the county level from the National Agricultural Statistical Service (NASS) to calculate the quantity and value of resources safeguarded by containing and in some cases, reversing the spread of wild pigs. The data on acreage, yields, and prices were sourced from the 2017 Census of Agriculture. The agricultural census is conducted every five years and the analysis focuses on the period spanning 2014 to 2021; thus, we assume that crop data from 2017 are constant across all years of the analysis. Further, information on livestock counts was obtained from the 2017 Census of Agriculture; however, data covering the annualized values of livestock are not available from NASS, which prevented us from investigating the value of livestock that were unthreatened by the spread of wild pigs. Lastly, data gathered from the 2015 USGS National Land Cover Database were used to estimate the acreage of wetlands, which would have been threatened by the unmitigated spread of wild pigs. To calculate the resource acreage that would have been exposed to wild pigs, we interact the total acreage in each county with the forecasted proportion of the county occupied. Dollar values for crops and land were also obtained for the year 2017 and were used to quantify the value of resources safeguarded by the NFSDMP. After estimating the annualized value of resources that would have been threatened by the spread of wild pigs, the total value of resources safeguarded was computed by aggregating across the additional years in which a county was forecasted to be occupied. 

### 2.2. Boosted Regression Tree Models

We estimated the covariate effects on response data using boosted regression trees with least-square boosting because the response variable did not follow a known parametric distribution and because we expected complex non-linear relationships between the response and covariates. Covariates for spread rates based on the difference in proportion of area occupied include the proportion occupied in the focal county in the previous year, the mean proportion of counties occupied in the adjacent counties (i.e., an occupancy pressure from adjacent counties), amount of wild pigs removed per km^2^ in the focal county in the previous year, the mean count of removals per county in the adjacent counties in the previous year (log scale), the proportion of the focal county with active removal work in the previous year, the mean proportion of adjacent counties with active removal work in the previous year, the habitat suitability index (the habitat suitability index standardizes biotic and abiotic factors across counties and is obtained using the methods of Ref. [18] for use as a covariate to fit the boosted regression tree models) [18], median density estimates rescaled, the percentage of the focal county with hydrology, the percentage of the focal county with tree cover, the percentage of the focal county with crops, the percentage of the focal county with pasture, and the percentage of deciduous plant cover.

Covariates for spread rates based on the difference in the area (km^2^) occupied in the focal county in the previous year, the mean proportion of counties occupied in the adjacent counties, the sum of removals in the focal county in the previous year, the mean count of removals per county in the adjacent counties in the previous year (log scale), the proportion of the focal county with active removal work in the previous year, the mean proportion of adjacent counties with active removal work in the previous year, the habitat suitability index [18] median density estimates rescaled, the percentage of the focal county with hydrology, the percentage of the focal county with tree cover, the percentage of the focal county with crops, the percentage of the focal county with pasture, and the percentage of deciduous plant cover. Several covariates are subject to the sources of error discussed in the previous section. Further, it is difficult to account for how wild pig management would have evolved in the absence of the NFSDMP in response to the continuously spreading wild pigs.

For each response, we fit models with and without the covariate describing status of the focal county in the previous year because this covariate was a very strong predictor. We took this approach to account for the extent of path dependency and uncertainty in forecasting the spread of wild pigs. When interpreting the results, it is important to emphasize that regardless of our modeling assumptions, the expansion of wild pig populations without intervention is considerable. We denote the first four models A through D for each possible response–covariate pair. Thus, we examined the following four models of wild pig spread: change in proportion occupied with proportion occupied in 2008 (model A), change in proportion occupied without proportion occupied in 2008 (model B), change in km^2^ with km^2^ occupied in 2008 (model C), and change in km^2^ without km^2^ occupied in 2008 (model D).

We use the fitted models to conduct step-ahead forecasting for years 2014–2021 (post-introduction of the NFSDMP), starting with the data in 2013. For each year, we use the Persistence and Transient models to predict the rate of change for each county given the current year’s covariate values. We then add the change to the current value in each county (proportion occupied for A and B models, km^2^ for C and D models). For A and B models, we bound the predictions between 0 and 1; for C and D models, we bound the predictions between 0 and the upper maximum of the county area in km^2^. For each year, we recorded which counties became newly occupied and which counties became newly unoccupied. We used the updated proportion occupied values to update the mean proportion occupied in adjacent counties to be used in the next year’s prediction. 

### 2.3. Dynamic Occupancy Models

We modeled the occupancy dynamics using a two-step process. We modeled the occupancy rate within 8-digit watersheds using 12-digit watersheds as spatial replicates. We then used a stochastic framework to account for changes in the annual rate of introduction (invasion) and extinction due to stochastic processes. The dynamic occupancy models do not account for imperfect detection due to computational infeasibility. Covariates include a combination of annually time-varying and non-time-varying climate and land cover data obtained from Refs. [7,18]. In addition, the rate of invaded watersheds in the previous year and the distance to the nearest invaded watershed were also included. Based on previous work [6,7,18,19], we included the following non-time and time-varying covariates for predicting the rate of spatial spread.

Non-time-varying covariates include terrain ruggedness, predicted equilibrium density [18], and mast tree species richness. Time-varying covariates are shown in Table 1. 

All models include the rate of invaded watersheds in the previous year and the distance to the nearest invaded watershed. Initial model screening was conducted using projection predictive variable selection that allows for large sets of candidate models to be evaluated quickly. We evaluated 108,537 possible candidate models using all possible combinations for eight sets of variables that did not have a large covariance. From that initial screening, a reduced set of 127 candidate models was evaluated using leave-one-out cross validation. Predictive capacity of the final model was evaluated using Bayesian version of R-squared and LOO-adjusted R-squared. Model convergence was evaluated by comparing the between- and within-chain predictions for model parameters as well as bulk and tail effective sample size. Evaluation of the top model indicated some potential regionally varying effects. To address this, we evaluated models with uncorrelated random intercepts and correlated intercepts with random slopes to account for regional variation in invasion rates. We considered four regions—west, plains, Great Lakes, and east. The final Bayesian model predicts the probability of a watershed being invaded annually given the covariates included in the model. Bayesian models and model evaluation were conducted using the rstanarm (rstanarm version 2.21.3 was used to estimate the model. The most recent update can be found at https://cran.r-project.org/web/packages/rstanarm/index.html, accessed on 14 June 2023) package within the R computing environment. 

To predict the spread of wild pigs in each year from 2014 to 2021, we used step-ahead forecasting using a stochastic framework to account for changes in annual rate of invasion and extinction due to stochastic processes. To implement the simulation, we first predict the probability of invasion using the final Bayesian model for all uninvaded watersheds in year t using associated time-varying data for year t. This results in a probability of invasion for every uninvaded watershed. The number of invaded watersheds in year t was drawn from a beta distribution representing the range of observed invasion rates from 2004 to 2012. Watersheds are randomly assigned as invaded using the probability of invasion as a weight. The proportion of 12-digit watersheds within each 8-digit invaded watershed is updated and the distance to the nearest invaded watershed for all uninvaded watersheds is updated. In year t + 1, the probability of invasion and watersheds assigned as invaded was the same as in year t. Additionally, in year t + n, the number of watersheds with extinctions was drawn from a beta distribution representing the range of observed extinction rates from 2004 to 2012. This process of simulation was conducted until t + 8 (2021). The simulation was run 1000 times. Using these simulations, the median proportion of each 8-digit watershed invaded was calculated. These watershed-level proportions are used to estimate the proportion of each county invaded. 

### 2.4. Resources Safeguarded

To evaluate the economic impacts of preventing the spread of wild pigs, we begin by accounting for all resources that would have been threatened under each modeling scenario. The resources that would have been threatened without intervention by the NFSDMP are also referred to as resources safeguarded due to intervention by the program. We interact the proportion of each county invaded by wild pigs with total resource acreage and livestock inventories. The acreage and value of resources were computed and visualized using the R computing environment (R version 4.3.1 and ggplot2 version 3.4.4 were used to generate maps and calculate acreage and values). A variety of resources are included under the following broad categories: field crops, fruits, vegetables, tree nuts, field and grass seeds, forage, and hay, land (including pasture and wetlands), and livestock. Details for each category can be found in Appendix A Table A1.

Studying the period running from 2014 to 2021, we allow for variation in the amount of time wild pigs invade a given county and changes over time in the proportion of a county occupied for each scenario. As a result of the caveats discussed above, there is a high degree of uncertainty within each scenario. Thus, aggregating across each scenario results in a high level of uncertainty when calculating the quantity of resources threatened by wild pigs in the absence of the NFSDMP.

### 2.5. Value of Resources Safeguarded

To quantify the value of resources that would have been threatened in the absence of intervention by the NFSDMP, we utilize information on the value of an acre for each resource for which data are available and scale by the additional proportion of a county that is forecasted to be occupied by wild pigs in each year.

Information on dollar values for crops is obtained using national-level prices. We then combine the price with information on crop yields at the state level, which produces a state-level dollar value per acre for each crop. Information on the dollar value for pasture comes from cash rents, measured in dollars per acre. This is available for 42 states and at the national level; the national-level value is used in states where cash rents are unavailable. For details on the dollar values used, see Appendix A Table A2.

For each model, we computed the total amount of resources for which the threat from wild pigs is averted. We refrained from estimating the value of damages to resources; though surveys have been conducted to estimate the rate of damages from wild pigs in 11 southern states [8,9], extrapolating these results to other regions is impracticable. Employing data on prices and yields, we then calculated the total value of resources safeguarded from wild pig presence due to the introduction of the NFSDMP. We calculated the value of resources safeguarded for each county and year and then aggregated these values across all years and counties for each model. The resulting value is presented for each broad category of resources and is available for each individual resource. 

## 3. Results

### 3.1. Boosted Regression Tree Models

A summary of the data showed that there were 250 counties that became newly occupied between 2009 and 2013 (the five years immediately before the start of NFSDMP). This gives an average rate of invasion of 50 counties per year (250 counties/5 years). On average, the newly established populations in these counties occupied 447.5 km^2^ (95% CI: [309.2, 585.7] km^2^) equivalent to 0.17 proportion of the county (95% CI: [0.14, 0.21] proportion of the county). Also, 78 counties became newly unoccupied, giving a rate of 15.6 counties/year equivalent to 284.2 km^2^/year (95% CI: [115.4, 453.0] km^2^). 

After the NFSDMP was introduced in 2014, 260 counties became newly occupied between 2014 and 2021, giving an average rate of 32.5 counties per year (260 counties/8 years). On average, the newly established populations in these counties occupied 226.3 km^2^ (95% CI: [179.2, 273.4] km^2^), equivalent to 0.20 proportion of the county (95% CI: [0.16, 0.24] proportion of the county). Also, 343 counties became newly unoccupied, giving a rate of 42.9/year equivalent to 166.0 km^2^/year (95% CI: [120.4, 211.5] km^2^). Thus, the rates of spread in terms of both the number of counties per year and the number of km^2^ per county per year were higher before the program started (the NFSDMP worked to unify federal, state, and local wildlife managers as well as landowners and university collaborators to manage wild pigs. A variety of approaches including but not limited to population control as well as eradication were used in reducing the spread of wild pigs after 2014). The rate of elimination in terms of the number of counties per year where wild pigs were eliminated was almost three times higher after the program began, but the area in each county where wild pigs were eliminated tended to be lower after the program relative to before the program started (166 km^2^/year vs. 284.2 km^2^/year).

**Figure 1:**
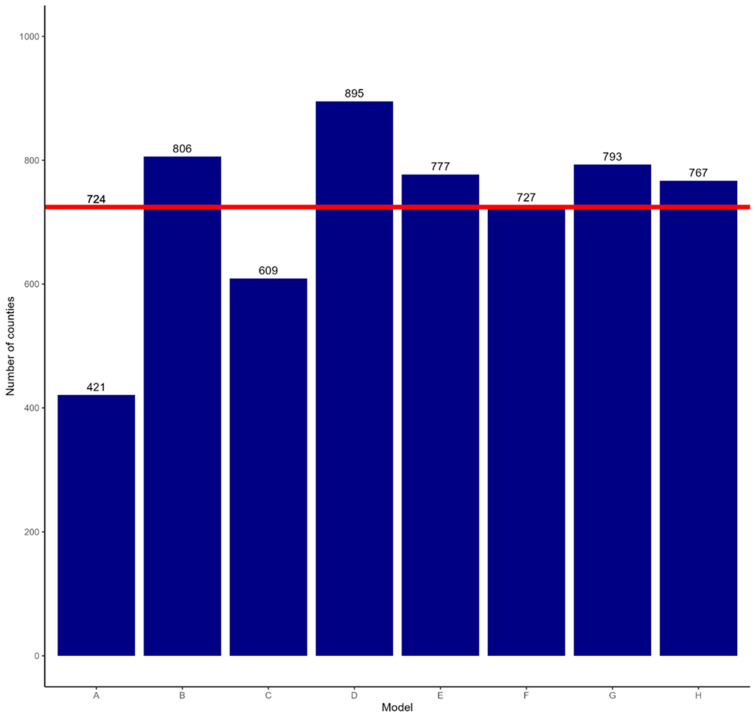
Number of counties forecasted to be newly occupied by wild pigs across all scenarios in the absence of the NFSDMP from 2014 to 2021. Across all frameworks, an average (red line) of 724 new counties are forecast to be occupied by wild pigs.

**Figure 2:**
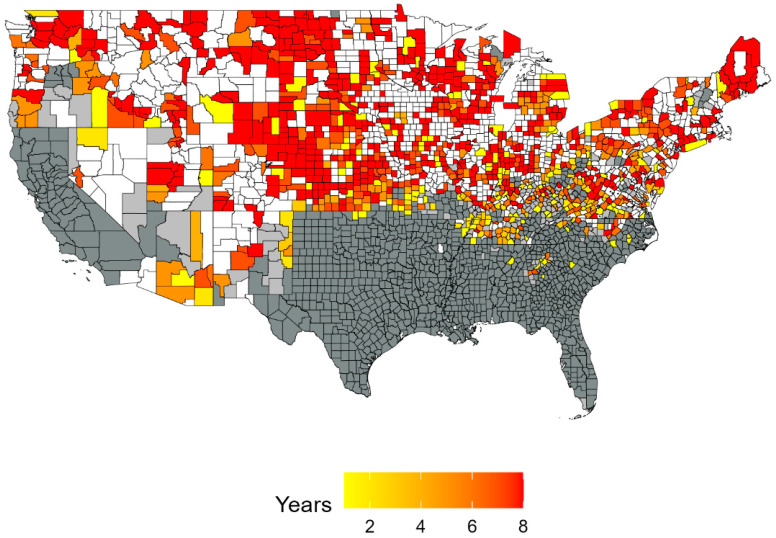
Counties where wild pigs are forecasted to spread under the assumptions of model D. This forecast is the most aggressive outcome. Dark gray counties were always occupied, light gray counties were partially occupied, and white counties were never occupied. Counties are forecasted to be occupied for as few as one additional year (yellow) up to eight additional years (red). Significant geographic variation is forecasted across other models.

The forecasted annual dynamics of number of counties newly occupied and unoccupied for model A matched the pre-program rates most closely (~55 counties newly occupied/per year and 12 counties newly unoccupied/year) (Figure 1). Models C and D forecasted much higher rates for both becoming newly occupied or unoccupied than the rates before the program (Figure 1). For example, model D forecasted the most aggressive outcome with up to 895 newly occupied counties (Figure 2).

### 3.2. Dynamic Occupancy Models

#### 3.2.1. Data Fitting

The final Bayesian statistical model includes six predictors—terrain ruggedness, Lewis density (the Lewis density is the predicted density of wild pigs from Ref. [18]), annual temperature range, annual mean precipitation, rate of invaded watersheds in the previous year, and the distance to the nearest invaded watershed. 

Evaluating models with only fixed effects, regional uncorrelated random intercepts, and regional random slopes with correlated intercepts found, all had generally good predictive performance. Further posterior predictive checks found no bias in the predictive capacity of the models. For detailed methods and results on the performance of the watershed scale models, see Appendix B.

#### 3.2.2. Forecasting

The median forecasted spread of wild pigs varied among models ranging from 1999 to 2065 watersheds invaded by the year 2021 (lower and upper 95-percentiles are included in result files). The number of invaded watersheds annually varied due to both annual time-varying predictors of invasion as well as stochasticity in invasion as well as extinction. Additionally, there were differences in the geographic patterns of invasion. In general, forecasts resulted in generally small proportions of the invaded counties being occupied by wild pigs.

Across all models, an average of 724 additional counties would have been occupied by wild pigs in the absence of intervention. This reflects an overall high degree of transience in wild pig populations, as 1272 counties were observed to be occupied by wild pigs in 2013. In the most conservative forecast, 421 counties are forecast to be invaded by wild pigs, and in the most aggressive forecast, 895 counties are occupied by wild pigs. Details regarding the number of counties invaded for each year forecasted can be found in Appendix A Table A3.

### 3.3. Resources Safeguarded

**Figure 3:**
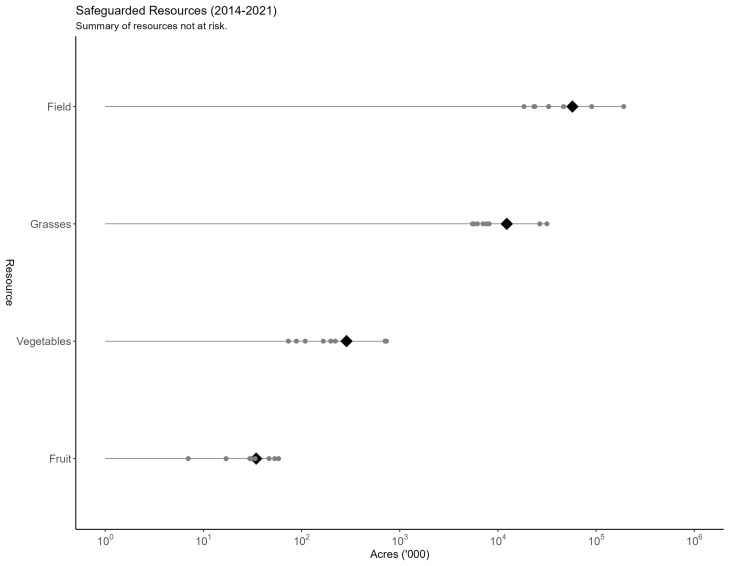
Summary of field crop acreage safeguarded from additional wild pig presence. Gray points represent each model forecast of resource acreage and black diamonds represent the mean acreage for each field crop. Acreage is scaled logarithmically. Acreage is cumulative over the period spanning 2014 to 2021.

There is wide variation across models in the quantity of resources safeguarded from wild pigs by the NFSDMP (see Figure 3). Taking an unweighted mean across each model, we find that an average of 60.12 million acres (243.3 thousand km^2^) of field crops, 13.43 million acres (54.4 thousand km^2^) of grasses and hay, 14.32 million acres (58 thousand km^2^) of wetlands, 118.9 million acres (481.2 thousand km^2^) of pasture, and 37.99 million heads of livestock would have been threatened by new wild pig populations without the NFSDMP. Further, the most significant crops that would have been threatened by wild pigs include 21.86 million acres (88.5 thousand km^2^) of corn, 21.22 million acres (85.9 thousand km^2^) of soybeans, and 11.84 million acres (47.9 thousand km^2^) of wheat.

**Figure 4:**
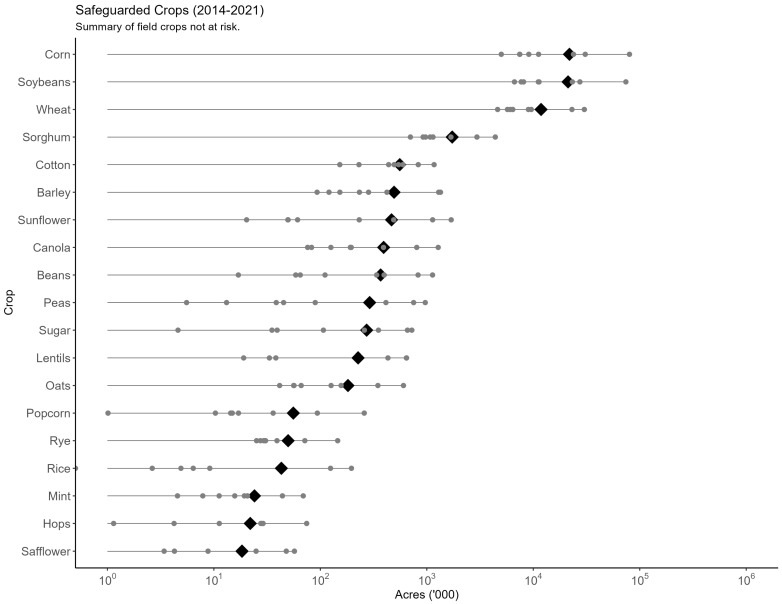
Summary of field crop acreage safeguarded from additional wild pig presence. Gray points represent each model forecast of crop acreage and black diamonds represent the mean acreage for each field crop. Acreage is scaled logarithmically. Acreage is cumulative over the period spanning 2014 to 2021.

**Figure A1:**
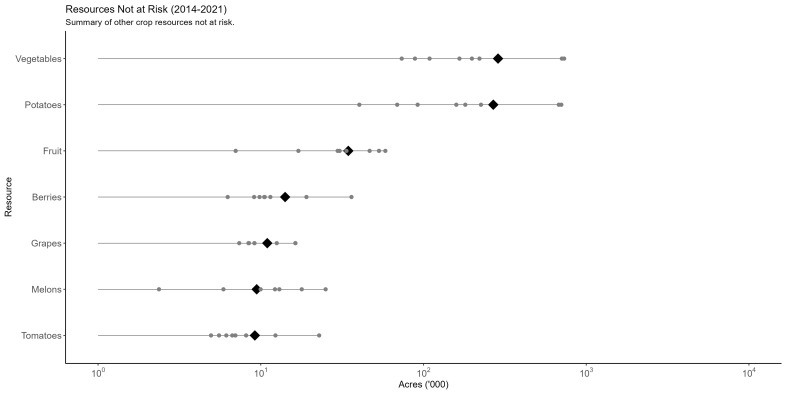
Summary of fruit and vegetable acreage safeguarded from additional years of wild pig presence. Gray points represent each model forecast of fruit and vegetable acreage and black diamonds represent the mean acreage for each fruit and vegetable resource. Acreage is scaled logarithmically. Acreage is cumulative over the period spanning 2014 to 2021.

**Figure A2:**
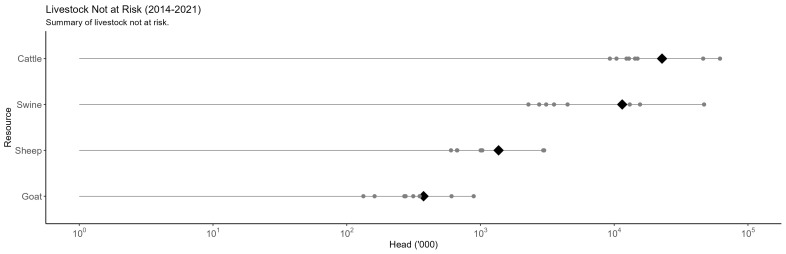
Summary of head of livestock safeguarded from additional wild pig presence. Gray points represent each model forecast of head of livestock and black diamonds represent the mean head of each type of livestock. Head of livestock is scaled logarithmically. Head of livestock is cumulative over the period spanning 2014 to 2021.

**Figure A3:**
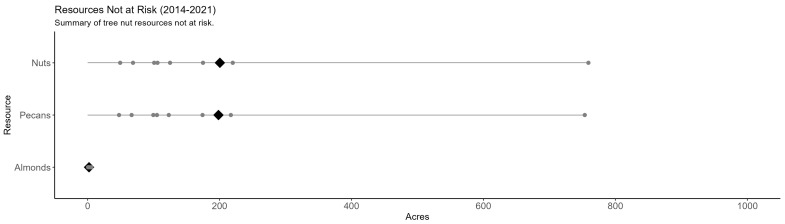
Summary of tree nuts safeguarded from additional wild pig presence. Gray points represent each model forecast of nut acreage and black diamonds represent the mean acreage for each type of tree nut. Acreage is scaled logarithmically. Acreage is cumulative over the period spanning 2014 to 2021.

**Figure A4:**
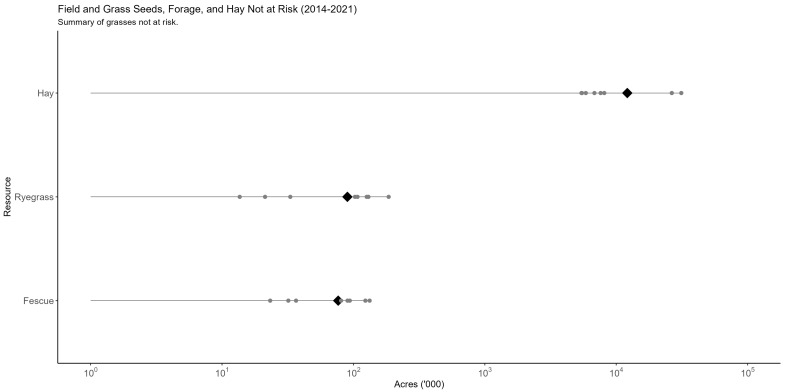
Summary of hay and grasses safeguarded from additional wild pig presence. Gray points represent each model forecast of hay and grass acreage and black diamonds represent the mean acreage for each type of hay or grass. Acreage is scaled logarithmically. Acreage is cumulative over the period spanning 2014 to 2021.

Figure 4 plots all model outcomes for the quantity of field crops threatened by wild pigs without intervention. In the most conservative estimates, as few as 17.91 million acres [72.5 thousand km^2^] of field crops and, in the most extreme estimate, as many as 198.3 million acres [802.5 thousand km^2^] of field crops would have been threatened by the presence of wild pigs without the implementation of the NFSDMP. The high level of uncertainty in the estimates of field crops threatened by wild pigs extends to additional resources. Appendix A Figure A1, Figure A2, Figure A3 and Figure A4 plot all model outcomes for the quantity of other resources safeguarded. For example, the field and grass seed, forage, and hay acreage threatened by the potential spread of wild pigs ranges from 5.61 to 33.62 million acres [22.7 thousand km^2^ to 136.1 thousand km^2^]. Further, the threat from wild pigs to pasture range from 37.06 to 319.4 million acres [150 thousand km2 to 1.29 million km^2^], wetlands range from 4.96 to 34.91 million acres [20.1 thousand km^2^ to 141.3 thousand km^2^], and livestock range from 12.05 to 117.4 million heads of livestock. This uncertainty is generated by sources ranging from the spread modeling assumptions, the speed at which wild pigs are forecast to spread, and the extent of and specific locations where wild pigs are forecast to invade.

### 3.4. Resources-Value of Resources Safeguarded

**Figure 5:**
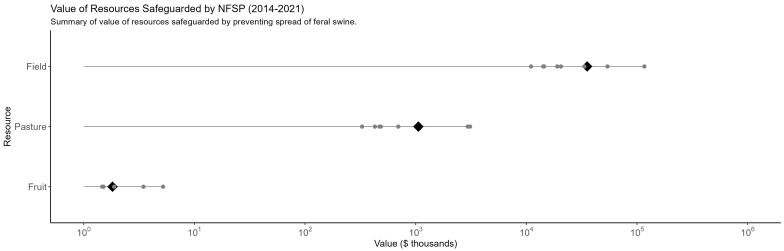
Summary of the value of resources safeguarded. The figure shows resource values for broad categories of resources. Gray points represent each model forecast of resource value safeguarded and black diamonds represent the mean value for each category of resource. Resource value is scaled logarithmically. Values are cumulative over the period spanning 2014 to 2021.

For a subset of resources, we use prices and rental rates to quantify the value of resources that were avoided by wild pigs through the implementation of the NFSDMP. The assumption that crop acreage and value from 2017 can be extrapolated for the entire period spanning 2014 to 2021 continues to be utilized to quantify the value of resources safeguarded by preventing the spread of wild pigs. The results indicate that between USD 11 and USD 116 billion in value of field crops, grasses, and pasture have been safeguarded between 2014 and 2021 (Figure 5). Due to a lack of robust estimates for the economic value of wetlands and annualized values for livestock, the analysis of the value of resources focuses on crops, grasses, and pasture. 

**Figure 6:**
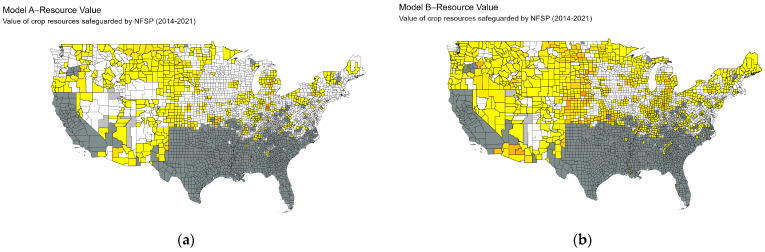

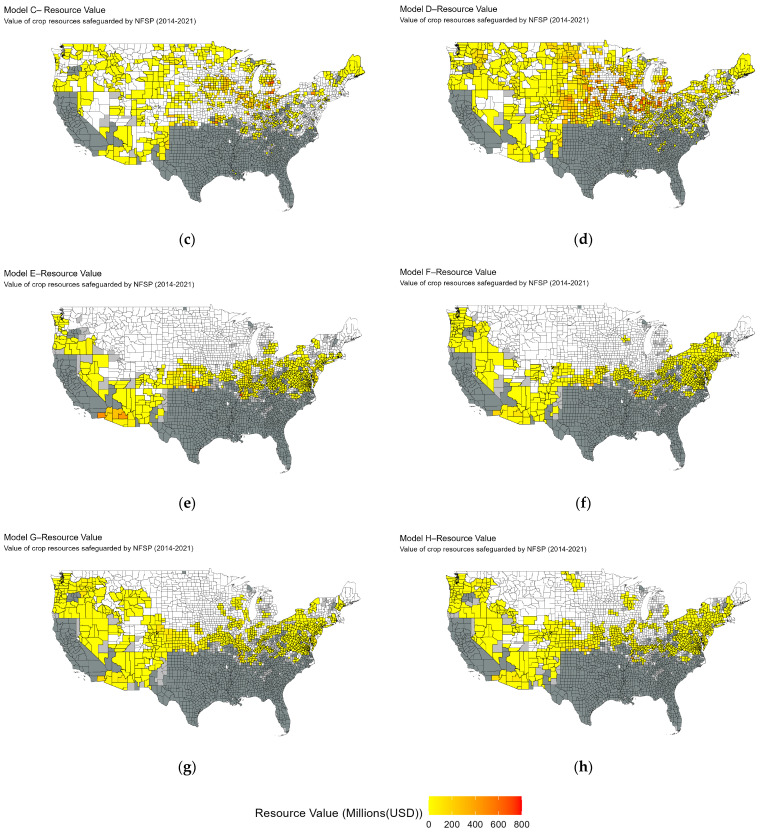
Spatial distribution of the value of resources threatened for each model. Each subfigure presents the value of resources safeguarded for one of eight model forecasts. Counties that are forecasted to be occupied by wild pigs are colored based on the amount of resource value safeguarded from lowest (yellow) to highest (red). Dark gray counties were always occupied, light gray counties were partially occupied, and white counties were never occupied and are not included in forecasting the value of resources safeguarded. Each model forecasts a large area that wild pigs would have occupied; however, in models C and D, wild pigs are forecasted to spread quickly to counties where many valuable resources are harvested, driving the large magnitudes that we estimate.

**Figure 7:**
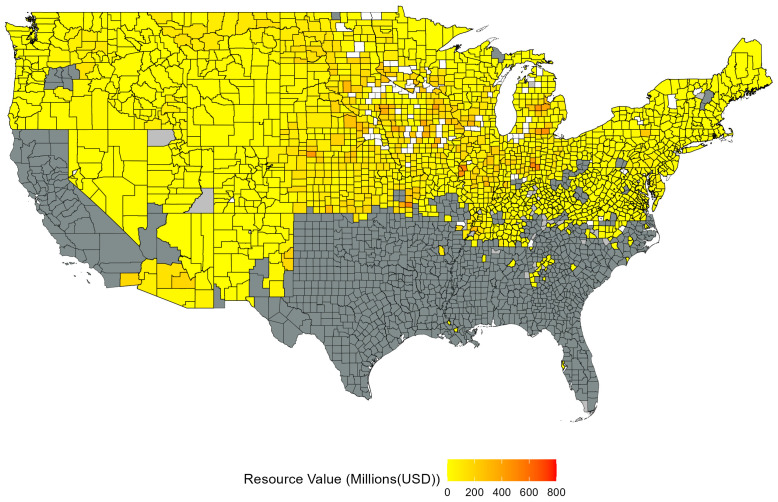
Average value of resources safeguarded across all models. In each model, counties that are never forecasted to be invaded by wild pigs are treated as zero. Counties that are forecasted to be occupied by wild pigs are colored based on the average amount of resource value safeguarded from lowest (yellow) to highest (red). Dark gray counties were always occupied, light gray counties were partially occupied, and white counties were never occupied and are not included in forecasting the value of resources safeguarded. The value of resources safeguarded is averaged across years for the period spanning 2014 to 2021.

Figure 6 maps the spatial distribution of the value of crops, grasses, and pasture precluded from wild pigs across US counties for each model. Figure 7 maps the mean value of crops, grasses, and pasture resources for each county across all models of wild pigs spread, cumulated across the period from 2014 to 2021. The spatial distribution indicates that a key factor in determining the value of resources safeguarded is the specific counties where wild pigs are predicted to spread. For example, the forecasted spread in model D encompasses areas of states with significant agricultural production, such as Nebraska, Iowa, and Illinois, while the forecasted spread in model F indicates that wild pigs spread to counties in eastern states with lower levels of agricultural production.

## 4. Discussion

Areas in which invasive wild pig populations have been established or may emerge are currently subject to outreach and eradication efforts under the NFSDMP. Our results indicate that in addition to these areas, these efforts by the NFSDMP have effectively prevented the spread of wild pigs to counties where wild pig populations have not been observed. We demonstrate that between 421 and 895 counties would have experienced at least one additional year of wild pig presence. For each additional year in which wild pigs were prevented from occupying a county, we compute and aggregate the acreage of land resources and estimate the value of a subset of commodities safeguarded. This analysis shows that between USD 11 and USD 116 billion in value of field crops, pasture, grasses, and hay has been safeguarded because of intervention by the NFSDMP between 2014 and 2021. This value does not include the value of resources protected in counties that have experienced wild pig presence throughout the study period. These results indicate that the introduction of the NFSDMP delivered significant economic benefits to regions that have not experienced a wild pig invasion. When evaluating the efficacy of the NFSDMP and other similar programs, these benefits must be considered. Further, if control efforts are maintained over time, the benefits of safeguarding resources will continue to grow as wild pig invasions are prevented over a longer timeframe. Regardless, the large range of outcomes with respect to the area experiencing additional wild pig presence and the economic value of resources safeguarded can be attributed to high levels of uncertainty present in the underlying modeling framework and several limitations, which are outlined below. Despite this uncertainty, without relying on a single most accurate model, the models collectively suggest that the magnitude of our results is large and significant.

Uncertainty regarding the spread of wild pigs and the extent of the damage resulting from wild pig presence is an additional challenge. The environmental and climatic factors that drive wild pig population growth documented in McClure et al. [19] have been influential in predicting their northward expansion [6]. Once established, Miller et al. [5] used watershed-scale modeling to uncover the determinants of wild pig population growth. Regardless, predicting the spread and growth of wild pig populations using ecological modeling utilizes pre-period data and a variety of assumptions. Due to errors in pre-period data, unpredictability in the ecological factors governing wild pig population dynamics, and assumptions inherent to a given modeling strategy, there is a high degree of uncertainty regarding the timing and location of wild pig population growth. Further, information on population densities in observed wild pig habitats is difficult to ascertain, which yields an additional source of uncertainty in spread forecasting. Lastly, the pre-period data used to inform the ecological models did not account for the international expansion of invasive wild pigs from Canada or Mexico. Notwithstanding, in all models we evaluated, the extent of the spread of wild pigs and the magnitude of the associated resources safeguarded are considerable.

There are significant challenges in ecological forecasting that result in potentially large uncertainties in predicted ecological states. To predict the spread of wild pigs in the contiguous US in the absence of the NFSDMP, we use a conservative approach with multiple modeling frameworks. This was performed to account for potential uncertainties and a diversity of different model assumptions. This has the benefit of providing multiple predictions that can be used in an ensemble framework for further assessment of potential economic impacts averted because of the NFSDMP. 

For each boosted regression tree model, we acknowledge the following caveats. First, the predictions are deterministic. Thus, in the first year of forecasting, most counties become newly occupied and the number decreases thereafter. This is because the average spread rate from 2009 to 2013 is quite high. We tried adding noise by sampling the predictions from a normal distribution using the variance of the empirical distribution. This results in more spread overall). Second, management occurred before the program, but it is difficult to predict how that would continue as new counties become invaded. We tried adding a separate model in the forecasting as follows. We updated the amount of removal in the focal county using a separate model of the relationship between removal amount per km^2^ or proportion of the county in the focal county in the current year and covariates (current km^2^ occupied, removal amount per km^2^ in the previous year, mean removal amount in adjacent counties in the previous year, and the habitat suitability and landcover variables as in the other models. We fit this model using the same methodology as the Persistence and Transient models, although we did not subset the data because there was no evidence that the relationship between take amount and covariates differed depending on historical wild pig presence. This model had an R^2^ of 0.67 and 0.94 for in-sample predictions of models of number removed/km^2^ and log numbers removed, respectively. At each time step, we used this model to update the amount of removal occurring in focal and adjacent counties in the previous year based on updated occupancy. This allowed us to maintain pre-program levels of removal as the spread to new areas progressed, but there is uncertainty in those estimates. These models produced very similar forecasts to their corresponding models without removal updates, except with slightly higher invasion rates overall). Lastly, there is a substantial amount of reporting variation both over time and space in the observed data. There are no data to explain it. Thus, our predictions are influenced by these data limitations. 

The following caveats apply to the dynamic occupancy models. Firstly, while we accounted for time-varying factors influencing the probability of invasion and stochastic processes of invasion and extinction, there is uncertainty about which model might represent the invasion process best. We also did not explore non-linearities among covariates. Furthermore, watershed-level models did not explicitly account for control that likely would have continued to occur without a national program, although they did allow for extinction, which may have resulted from local or regional control activities. We assume that regional effects (random intercept and slopes) account for some of this variation. Additionally, associations with covariates are likely influenced greatly by the geographic location of the spread that occurred from 2004 to 2012. The majority of spread occurred in more arid regions and likely influenced covariate associations. This could be addressed by fitting models to different subsets of data. Finally, we assume perfect detection of watersheds with wild pigs, which is likely invalid. 

Several caveats limit the scope of the economic analysis as it pertains to valuing the resources avoided by wild pigs due to the NFSDMP. First, comprehensive estimates of the annualized value of wetlands and livestock are not available to be used in the analysis. Specifically, previous evaluations of wetlands [20] have examined specific sites and estimated the value of specific features of wetlands; however, these estimates have not been applied to all wetland acres throughout the United States. Additionally, estimates of the stock value of livestock have been produced for cattle, domestic swine, sheep, and goats [21,22]; however, we are not aware of studies that convert these to annualized values that account for the production and depreciation of livestock. Lastly, Brown et al. [21] and Sauter-Louis et al. [23] demonstrate the extent of disease transmission risk from wild pigs to livestock, emphasize the degree of uncertainty regarding the economic consequences of disease risk, and highlight the self-sustaining cycles of disease transmission between wild pigs and domestic livestock. For example, the costs resulting from the death of livestock, culling exposed livestock, and potential trade restrictions due to a disease outbreak [24,25] have not been precisely estimated. For the above reasons, we focused primarily on field crops, fruits and vegetables, and pasture for the economic analysis.

Second, we completed our analysis under the assumption that resource acreage and prices would have been unaffected by the presence of wild pigs. In counties where the NFSDMP reduced or prevented wild pig populations from being established, it is possible that had wild pigs been present, producers would have responded by changing the mix of crop and livestock resources from what was observed in the 2017 Census of Agriculture. A reduction in planted crop acreage would have reduced the quantity of resources threatened by wild pigs; however, if this reduction would have generated an increase in price then the value of resources safeguarded would have increased. With limited information regarding changes in acreage and prices that would have resulted from a wild pig invasion, the effect of this assumption is ambiguous.

Lastly, the analysis assumed that the only way to control, mitigate, and eradicate wild pig presence and damage was through the NFSDMP. In the scenario where the NFSDMP was never introduced, it is likely that producers and landowners would have privately attempted to limit the spread of wild pigs. Further, we have ignored efforts that could have replicated the NFSDMP at the state and county levels. Though it is likely that effort would have been made to mitigate the impact of the spread of wild pigs in the absence of the NFSDMP, it is difficult to analyze the costs of implementing these measures at the sub-national level as well as their effectiveness in the absence of NFSDMP intervention. Acknowledging the above, interventions in the absence of the NFSDMP would have reduced the probability of a wild pig invasion relative to the model forecasts, and we are unable to account for this.

## 5. Conclusions

Our findings indicate that in the absence of intervention by the NFSDMP, invasive wild pig populations would have likely continued to spread widely across the United States. Various modeling scenarios demonstrate that there is large uncertainty regarding the exact locations where wild pigs would have spread. Regardless, across each scenario, we find that significant resources have been safeguarded due to intervention by the NFSDMP. As few as 421 and as many as 895 counties are invaded across the various model scenarios. Taking the average across each scenario, we show that an additional 60 million acres of field crops, 13 million acres of grasses and hay, 14 million acres of wetlands, 119 million acres of pasture, and 38 million heads of livestock would have been threatened by the spread of wild pigs in the absence of the NFSDMP. Lastly, we calculate that the value of field crops, grasses and hay, and pasture safeguarded from the spread of wild pigs by the NFSDMP is on average USD 40.2 billion over the period from 2014 to 2021.

These findings indicate that the economic benefits of introducing the NFSDMP spread far wider than previously described, although a significant portion of funding and resources provided by the NFSDMP has been focused on areas where wild pigs are already known to be present or have continued to impose damages to resources and property. A less commonly known outcome of the NFSDMP is the prevention of the spread of wild pigs and the associated threat to resources in regions where invasive feral swine populations may have emerged. The economic benefits that flow to these areas as a result should be considered as a component of the comprehensive benefits of introducing the NFSDMP. Future work that evaluates the effects of wild pig management should take into consideration the findings of this paper.

## Figures and Tables

**Table 1:** Time-varying covariates used to predict spread in watershed-scale models. Land cover and indexed data are provided at an annual scale; thus, other covariates are also measured as an annual average.

Climate (Annual Average)	Land Cover (Annual Average)	3-Year Moving Average
Minimum Temperature Precipitation Snow Cover	Human Population Density	Enhanced Vegetation Index
	Agriculture (proportion)	Palmar Drought Severity Index
	Forest (proportion)	Minimum temp.
	Grassland (proportion)	Precipitation
	Wetland (proportion)	Snow cover
	Enhanced Vegetation Index	Enhanced Vegetation Index
	Palmar Drought Severity Index	

## Data Availability

The presence/absence of wild pig data presented in this study are available on request from the corresponding author due to privacy issues related to the National Feral Swine Mapping System. Crop acreage and values are openly available from https://www.nass.usda.gov/Quick_Stats/. The data was accessed on 29 November 2023.

## Appendix A. Tables

**Table A1:** List of all resources analyzed, grouped by six broad categories.

Field Crops	Vegetables	Fruit and Tree Nuts	Field and Grass Seeds, Forage, and Hay	Land	Livestock
Corn	Melons	Almonds	Hay	Wetlands	Cattle
Wheat	Potatoes	Pecans	Fescue	Pastureland	Swine
Sorghum Peanuts Rice Soybeans Sugar Cotton	Tomatoes	Grapes Berries	Ryegrass		Sheep Goat
Oats Barley Tobacco Beans Peas Flaxseed Lentils Hops Millet Mint Popcorn Rye Safflower Sunflower					

**Table A2:** National-level dollar values used to calculate county level value of resources safeguarded for a subset of resources.

Resource	Value (USD)
Corn	3.36/bushel
Wheat	4.44/bushel
Sorghum	3.05/bushel
Peanuts	0.21/lb
Rice	11.2/cwt
Soybeans	9.39/bushel
Sugar	31/ton
Cotton	0.72/lb
Cantaloup	19.20/cwt
Honeydew	28/cwt
Watermelon	14.70/cwt
Almonds	2.50/lb
Pecans	1.75/lb
Grapes	881/ton
Hay	137/ton

**Table A3:** Total counties forecasted to be occupied by wild pigs for each model and year.

Year	A	B	C	D	E	F	G	H
2013	1272	1272	1272	1272	1272	1272	1272	1272
2014	1473	1841	1633	1970	1485	1437	1474	1481
2015	1540	1890	1797	2090	1638	1594	1574	1623
2016	1558	1933	1754	2115	1708	1686	1747	1771
2017	1616	2055	1801	2154	1819	1785	1844	1842
2018	1651	2056	1861	2165	1871	1839	1883	1883
2019	1655	2059	1794	2131	1940	1884	1964	1956
2020	1676	2076	1858	2171	2011	1954	2013	2024
2021	1693	2078	1881	2167	2049	1999	2039	2065

## Appendix B

For the county-scale models, we estimated hyperparameters using Bayesian optimization with uniform prior distributions and mean squared error minimization as the criteria for parameter acceptance. The prior ranges were [50, 2000] for the number of learning cycles, [0.0001, 0.1] for the learning rate, [5 (Persistence models) or 10 (Transient models), 100] for the minimum leaf size, and [1, 50] for the maximum number of splits. We examined the fit of the models using R-squared (squared correlation of the observed and predicted response data). The fits of the models for each of the persistence (P) and transient (T) models with their respective most important predictors were as follows:

Model A: Change in proportion occupied with proportion occupied in 2008: P—0.83, T—1.0
P: Proportion occupied in t − 1, Pasture cover, Proportion of adjacent counties worked in t − 1
T: Proportion occupied in t − 1
Model B: Change in proportion occupied without proportion occupied in 2008: P—0.55, T—0.74
P: Pasture cover, Proportion of adjacent counties worked in t − 1
T: Pasture cover, Removal in adjacent counties in t − 1
Model C: Change in km^2^ with km^2^ occupied in 2008: P—0.98, T—1.0
P: Pasture cover, Tree cover, Water cover, Deciduous, Removal in adjacent counties in t − 1
T: Proportion occupied in t − 1
Model D:Change in km^2^ without km^2^ occupied in 2008: P—0.88, T—1.0
P: Pasture cover, Tree cover, Water cover, Deciduous, Removal in adjacent counties in t − 1
T: Pasture cover, Crop cover, Removal in adjacent counties in t − 1

For the watershed-scale models, because there was uncertainty in the best model and visual evaluation indicated regional differences in predicted probability of invasion, we considered four models in our final simulations. These models included (a) fixed effects only, (b) uncorrelated random intercept, (c) random slope for ruggedness and Lewis density and a uncorrelated random intercept, (d) random slope for Lewis density with correlated intercept.

**Table A4:** Detailed model specifications and fit for models E, F, G, and H.

Model	ELPD Difference	Bayesian R^2^		LOO R^2^	LOOIC Weight	Performance Score
		Conditional	Marginal	Conditional		
ruggedness|region + lewis.density|region + 1|region	0.00	0.9632	0.9645	0.8775	3.2 × 10^−1^	64.4%
lewis.density|region	−31.47	0.9632	0.9601	0.8765	3.6 × 10^−7^	25.0%
lewis.density|region + 1|region	−31.76	0.9632	0.9602	0.8764	3.7 × 10^−2^	26.6%
ruggedness|region + 1|region	−85.91	0.9633	0.9658	0.8755	8.0 × 10^−6^	25.0%
ruggedness|region	−86.46	0.9633	0.9657	0.8756	2.0 × 10^−5^	25.0%
1|region	−179.18	0.9632	0.9607	0.8738	9.5 × 10^−2^	29.1%
fixed only	−7071.29	0.9840	0.9840	0.8249	5.5 × 10^−1^	50.0%
